# Biglycan Reconstitutes a Neonatal ECM Signaling Microenvironment to Drive Stem Cell-Mediated Tendon Regeneration via a Scaffold-Free Cell Sheet Platform

**DOI:** 10.3390/ijms27104380

**Published:** 2026-05-14

**Authors:** Wen-Tao Li, Jia-Kuo Yu, Guo-Qing Cui

**Affiliations:** 1 Institute of Sports Medicine, Peking University Third Hospital, Peking University Health Science Center, 38 Xueyuan Road, Beijing 100191, China; li_wentao@bjmu.edu.cn; 2 Institute of Orthopedic and Sports Medicine of Tsinghua Medicine, Tsinghua University, 30 Shuangqing Road, Beijing 100084, China

**Keywords:** biglycan, tendon stem/progenitor cells, tendon regeneration, cell sheet therapy, anisotropic tissue repair

## Abstract

Unlike newborns, tendon injuries in adults usually lead to fibrotic scarring rather than functional regeneration. This difference is primarily due to the loss of neonatal extracellular matrix (nECM) signaling in adulthood. In this study, we investigated the molecular mechanisms by which a key neonatal ECM proteoglycan, biglycan (Bgn), orchestrates the behavior of tendon stem/progenitor cells (TSPCs) within a scaffold-free 3D cell sheet microenvironment that recapitulates native tendon architecture. Through immunofluorescence screening, we confirmed that Bgn is the predominant proteoglycan in neonatal rat Achilles tendons. Functional validation showed that adding Bgn to cell sheet cultures promoted TSPCs proliferation, maintained stem cell properties, induced tendon differentiation, and encouraged anisotropic alignment—effects similar to those of intact neonatal ECM. Immunodepletion experiments confirmed the causal role of Bgn. Notably, transplanting Bgn-conditioned TSPCs sheets into a rat full-thickness Achilles tendon defect model significantly restored final tensile load, collagen maturation, and gait function. These outcomes were statistically indistinguishable from those of the uninjured contralateral limb. These findings confirm that Bgn-functionalized cell sheet therapy is a viable translational strategy that can effectively recreate a natural 3D regenerative microenvironment. This work sheds light on the mechanisms involved in the determination of stem cell fate by the ECM and establishes Bgn-functionalized cell sheet therapy as a translatable, scaffold-free strategy for overcoming fibrotic repair and restoring functional tendon architecture.

## 1. Introduction

As mechanical conduits connecting muscles to bones, tendons efficiently transmit contractile forces to joints under repetitive, high-amplitude, cyclic loads [1]. The unique structural basis for this biomechanical function lies in the layered organization of the tendon ECM. Type I collagen molecules self-assemble into fiber bundles with strictly regulated diameters. These bundles then aggregate into fibers, which align longitudinally to form fascicular structures. These structures are then enclosed by the inner tendon sheath and surrounded by the outer tendon sheath [2,3]. Embedded within this ordered matrix are spindle-shaped tendon cells and a population of tendon stem/progenitor cells (TSPCs), which maintain matrix homeostasis and coordinate responses to mechanical stimuli. It is this structure, rather than collagen content alone, that underpins the mechanical properties essential for tendon function.

Tendon injuries, including acute ruptures and chronic tendinopathies, represent a significant global burden on musculoskeletal health. The incidence of Achilles tendon ruptures ranges from 11 to 37 cases per 100,000 person-years, and this rate is increasing as participation in recreational sports rises [4]. Currently, surgical repair is the standard treatment for complete ruptures. However, it requires a lengthy postoperative rehabilitation period, and the re-rupture rate remains at 2–10%. Furthermore, a significant proportion of patients report persistent pain, muscle weakness, and functional limitations [5]. The underlying cause lies in biological mechanisms: healing in adult tendons is essentially reparative rather than regenerative. The wound is filled with a disorganized provisional matrix rich in type III collagen, fibronectin and tenascin-C. This matrix is structurally inferior to native tissue and susceptible to re-injury [6,7]. Histologically, this fibrotic scar tissue exhibits randomly oriented collagen fibers, circular fibroblasts and a significantly reduced type I/type III collagen ratio. These characteristics collectively indicate a poor long-term mechanical prognosis.

A comparison of tendon healing in fetuses and preterm infants is instructive. Multiple independent studies in sheep, rabbit and mouse models have shown that fetal tendons can heal without scarring, restoring near-normal collagen fiber structure, tendon cell morphology and biomechanical properties within weeks of injury [8,9]. Crucially, this regenerative capacity is not solely an intrinsic property of fetal cells: when fetal tendon tissue is transplanted into an adult wound environment, its regenerative behavior is partially preserved. Conversely, when adult tissue is transplanted into a fetal wound environment, it acquires certain regenerative properties [8]. These complementary transplantation studies suggest that the local ECM microenvironment primarily determines the regeneration versus repair dichotomy. This leads to the hypothesis that providing the molecular signals abundant in the nECM could redirect the adult healing process towards regeneration.

The ECM is not a passive structural scaffold. Rather, it is an active signaling reservoir capable of storing, presenting and releasing growth factors, proteoglycans and matrix proteins, which directly regulate the behavior of resident cells through integrin receptors, receptor tyrosine kinases and morphogenetic gradients [10]. During fetal and early postnatal development, the tendon matrix contains a unique array of small leucine-rich proteoglycans (SLRPs) and fiber-associated collagen proteins. However, the levels of these components decline sharply in adult tissues [11,12]. Indeed, growing evidence indicates that SLRPs play instructive roles beyond structural support, functioning as signaling molecules that can reconstitute a neonatal-like microenvironment to orchestrate cellular behaviors including proliferation, differentiation, and matrix organization. For example, decorin has been shown to modulate TGF-β signaling and improve viscoelastic recovery in aged tendon healing models, while fibromodulin deficiency leads to abnormal collagen fibril morphology and impaired tendon mechanical properties. These findings collectively establish that SLRP family members are critical determinants of tendon healing quality, providing the rationale for investigating biglycan as a key neonatal ECM (nECM) effector in the present study. This developmental shift coincides precisely with the transition of the healing paradigm from regeneration to repair, suggesting a functional link between matrix composition and the quality of repair. However, the specific molecular entities responsible for this process, as well as the cells and signaling mechanisms through which they exert their effects, remain unclear.

TSPCs are a resident population of cells with clonogenic capacity and multipotency. They were first characterized by Bi et al. in 2007 [13]. They express classic stem cell markers, including CD146, nucleostemin and Oct-4, and can differentiate into tendon, bone, cartilage and fat depending on the microenvironmental context [14]. Under healthy, steady-state conditions, TSPCs help to maintain the tendon matrix’s low turnover rate. Following injury, they are recruited to the injury site, where the local biochemical environment determines their fate: a tendon-generating microenvironment drives efficient matrix remodeling, whereas pro-inflammatory or mechanically abnormal environments cause TSPCs to undergo ectopic ossification, chondrogenic metaplasia, or fibrotic scarring [15]. Restoring the guiding microenvironmental signals that maintain the differentiation potential of tendon stem cells towards tendon formation, promote ordered collagen deposition and coordinate collective cellular organization is essential in the field of tendon regenerative medicine.

Biglycan (Bgn) is a SLRP encoded by the BGN gene. It is characterized by a central leucine-rich repeat (LRR) domain flanked by two glycosaminoglycan (GAG) binding sites that typically carry chondroitin sulfate or dermatan sulfate chains [16,17]. This protein’s core binds to collagen fiber bundles via its LRR domain, and its role in regulating fiber bundle diameter, fiber spacing and the mechanical properties of connective tissue has been extensively studied [18,19]. Bgn knockout mice exhibit reduced bone mineral density, disorganized collagen fiber alignment in tendons and ligaments, and a progressive connective tissue phenotype that replicates several features of Ehlers–Danlos syndrome [20]. In addition to its structural role, emerging evidence suggests that Bgn itself functions as a signaling molecule. Soluble Bgn, released from the ECM, can bind to Toll-like receptors 2 and 4, thereby regulating innate immune responses. Studies have also shown that Bgn interacts with components of the bone morphogenetic protein (BMP) and Wnt pathways. This makes Bgn a potential microenvironmental regulator; its expression is highest in neonatal tendons, gradually declining with postnatal maturation. This temporal pattern precisely correlates with the loss of regenerative capacity [6,21,22].

To investigate whether Bgn is a key bioactive determinant in the ECM of the neonatal tendon, we employed a stepwise experimental strategy. Using immunofluorescence localization techniques, we documented the spatial expression of Bgn and its postnatal decline. This confirmed that Bgn is a highly enriched proteoglycan in the ECM of the neonatal rat Achilles tendon. To determine whether Bgn alone is sufficient to replicate the proliferative and stem cell property-maintaining effects of the nECM, as well as its ability to form tendons and promote cell alignment, we added recombinant Bgn directly to TSPCs culture medium while simultaneously performing Bgn-specific immunodepletion on the nECM. This allowed us to establish the causal necessity of Bgn. Having confirmed that Bgn is the primary bioactive factor, we prepared and characterized TSPCs cell sheets cultured in Bgn-conditioned medium. We then transplanted these cell sheets into a rat full-thickness Achilles tendon defect model to evaluate their ability to guide ordered Achilles tendon regeneration and inhibit fibrotic scar formation in vivo.

## 2. Results

### 2.1. nECM Promotes TSPCs Bioactivity Across Multiple Functional Endpoints

In order to identify age-dependent differences in matrix-driven bioactivity, we first compared TSPCs that were cultured in media supplemented with either nECM or aECM. We then evaluated the same set of four functional endpoints that were subsequently used for mechanistic analysis: proliferation, stem cell properties, tenogenic differentiation and anisotropic alignment.

**Figure 1:**
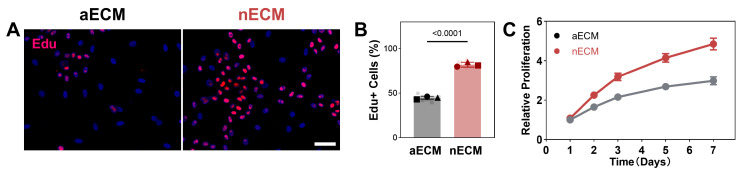
**nECM supplementation promotes TSPCs proliferation compared with aECM.** (**A**) Representative fluorescence micrographs of EdU incorporation (red) with DAPI nuclear counterstain (blue) in TSPCs cultured on aECM and nECM substrates. Scale bar = 200 μm. (**B**) Quantification of EdU^+^ rate (N = 3, *n* = 15). (**C**) CCK-8 kinetic proliferation assay over 7 days (*n* = 6).

Proliferation. Compared with aECM supplementation, nECM supplementation was found to significantly increase the EdU^+^ rate in TSPCs (*p* < 0.0001). This pattern was also evident in CCK-8 kinetic assays, in which the nECM group demonstrated a sustained proliferative advantage over the aECM group from days 3 to 7 (*p* < 0.0001). These results suggest that this proliferative effect is unique to nECM (Figure 1).

**Figure 2:**
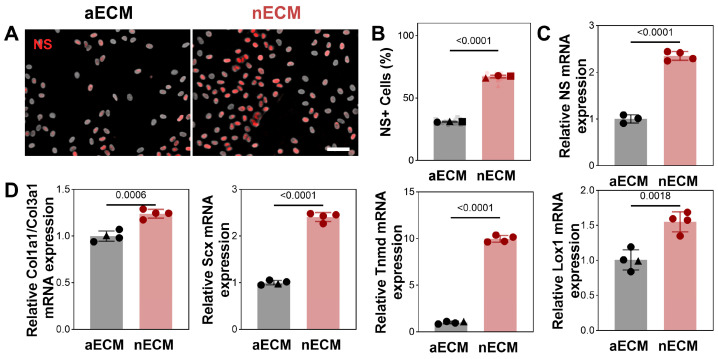
nECM supplementation maintains TSPCs stemness and promotes tenogenic differentiation. (**A**) Representative fluorescence micrographs of Nucleostemin (NS, red) and DAPI (grey) in TSPCs cultured on aECM or nECM substrates. Scale bar = 200 μm. (**B**) Quantification of NS^+^ cell proportions at day 7 (N = 3, *n* = 15). (**C**) NS mRNA expression at day 7 (*n* = 3 or 4; outliers excluded). (**D**) RT−qPCR quantification of Col1a1/Col3a1, Scx, Tnmd, and Lox1 mRNA expression at day 7. nECM supplementation significantly upregulated all tenogenic markers compared with aECM (*n* = 3 or 4; outliers excluded).

Stemness. Compared with the aECM group, the nECM group exhibited a notably higher percentage of NS^+^ cells by day 7 (*p* < 0.0001). The mRNA expression of NS+ cells followed a similar trend: supplementation with nECM significantly increased the expression of stem cell markers compared to aECM (*p* < 0.0001). These results suggest that nECM is more effective at maintaining the stem cell phenotype of TSPCs than aECM (Figure 2A–C).

Tenogenic Differentiation. RT-qPCR analysis after 7 days showed that nECM supplementation significantly increased the expression of Col1a1, Scx, Tnmd and Lox1 compared with aECM, thereby significantly raising the COL1A1/COL3A1 ratio. By contrast, aECM supplementation resulted in only slight upregulation of these tendon differentiation markers and a significantly lower COL1A1/COL3A1 ratio than in the nECM group (*p* < 0.0001) (Figure 2D).

**Figure 3:**
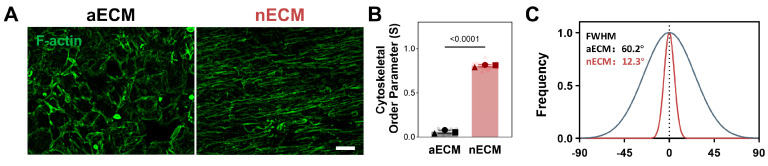
nECM supplementation promotes anisotropic TSPCs alignment under cyclic tensile strain. (**A**) Representative F-actin(green) fluorescence micrographs of TSPCs cultured on aECM or nECM substrates for 7 days. Scale bar = 200 μm. (**B**) Cytoskeletal Order Parameter quantification (N = 3, *n* = 15). (**C**) Directional distribution histograms of cell orientation.

Anisotropic Alignment. The cells were cultured for 7 days under cyclic uniaxial tensile strain (10%, 0.5 Hz, four hours daily). F-actin staining revealed that cells in the nECM-containing medium exhibited dense, parallel, longitudinal alignment. This was in stark contrast to the random distribution of cells in the aECM group. Quantitative orientation analysis confirmed that the coherence index was significantly higher in the nECM group than in the aECM group (*p* < 0.0001). Histogram analysis of directional distribution revealed a similar pattern, indicating that nECM uniquely promotes ordered collective cell alignment, whereas aECM lacks this ability (Figure 3).

Compared to aECM supplementation, nECM supplementation produced significant and consistent enhancements in TSPCs proliferation, stemness, tendon differentiation and anisotropic alignment across all four functional endpoints. These findings confirm that nECM possesses unique biological activity and suggest that the neonatal matrix is rich in molecular determinants absent from the adult matrix. Previous studies have confirmed that TSPCs reside in an ECM microenvironment in which biglycan and fibronectin (Fn) serve as key components and that Bgn depletion affects TSPCs differentiation via the BMP signaling pathway, impairing in vivo tendon formation [13]. Furthermore, Zhang et al. demonstrated that Bgn regulates the differentiation of tendon-derived stem cells (TDSCs) via the BMP7/Smad1/5/8 pathway. Based on these reports, we identified Bgn as a significant candidate molecule that is enriched in the nECM (see Section 3.2 for details). Subsequent experiments evaluating soluble Bgn supplementation tested whether this molecule alone could account for the broad spectrum of biological activity mediated by the nECM.

### 2.2. Bgn Is the Principal nECM Bioactive Molecule

Due to the significant biological activity of nECM across multiple functional endpoints, we aimed to identify the molecular determinants underlying this age-dependent effect. During tendon ECM maturation, proteoglycans undergo dynamic remodeling. In this process, Bgn—a SLRP that is abundant in developmental ECM—regulates cell-ECM interactions and promotes tissue regeneration. Based on these considerations, we hypothesized that Bgn is the primary bioactive driver of the nECM-mediated TSPCs-enhancing effects.

**Figure 4:**
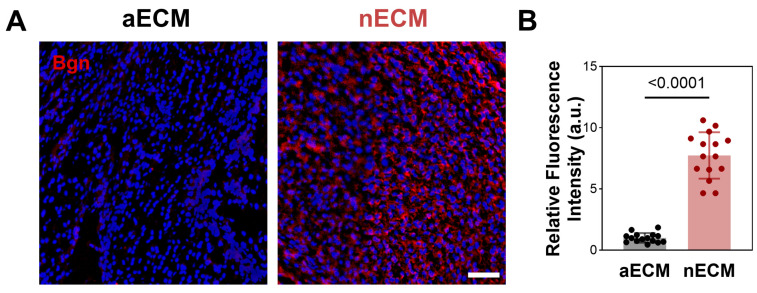
Bgn is abundantly expressed in neonatal rat Achilles tendon. (**A**) Representative immunofluorescence images showing Bgn distribution (red) in neonatal (P5) and adult (12-week) rat Achilles tendon cryosections, nuclei are counterstained with DAPI (blue). (**B**) Quantification of relative Bgn fluorescence intensity.

To validate this hypothesis, we examined the distribution of Bgn in frozen sections of freshly harvested Achilles tendons from neonatal and adult rats using immunofluorescence staining (Figure 4). Bgn exhibited strong immunoreactivity and was uniformly distributed in the interstitial spaces of neonatal rat Achilles tendons (P5); in contrast, immunoreactivity was significantly reduced in adult rat Achilles tendons (12 weeks). In summary, these findings confirm that Bgn is a highly abundant non-structural ECM component in the Achilles tendons of newborn rats, providing a theoretical basis for its role in mediating nECM-driven TSPCs enhancement.

Having identified Bgn as the primary candidate substance enriched in nECM, we then investigated whether adding soluble recombinant Bgn (2667-CM, R&D Systems) to the culture medium was sufficient to replicate the biological activity of nECM across four pre-specified TSPCs functional endpoints.

**Figure 5:**
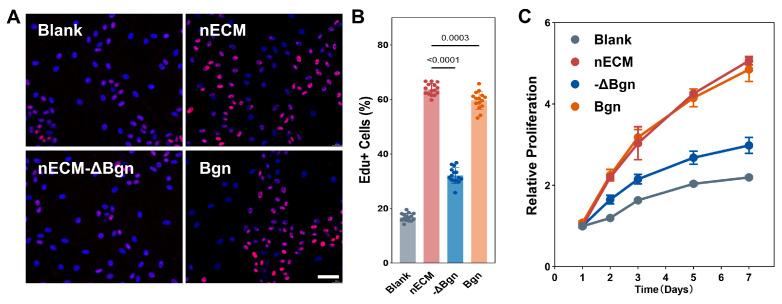
Bgn promotes TSPCs proliferation comparably to nECM. (**A**) Representative EdU incorporation images of TSPCs cultured under Blank, nECM, nECM−ΔBgn, and Bgn conditions; EdU (red), DAPI (blue). Scale bar, 200 μm. (**B**) Quantification of EdU+ cells (%) (*n* = 15). (**C**) CCK-8 proliferation curves over 7 days (*n* = 6).

Proliferation. The proliferation activity in the Bgn (1 μg/mL) group was statistically equivalent to that in the nECM (50 μg/mL) group and was significantly higher than that in the solvent control group (*p* < 0.0001). CCK-8 kinetic assays confirmed that, from day 3 onwards, the Bgn and nECM groups exhibited a proliferative advantage over the solvent control group, which persisted until day 7 (*p* < 0.001). This indicates that they provide sustained support for cell growth (Figure 5).

**Figure 6:**
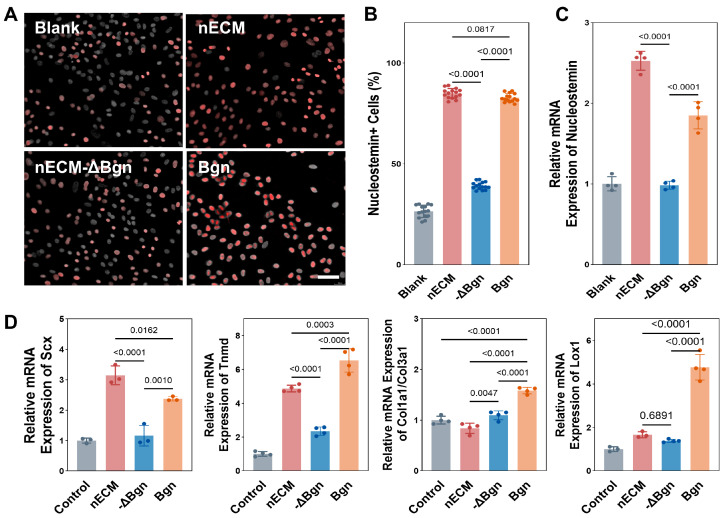
Bgn maintains TSPCs stemness and promotes tenogenic differentiation equivalently to nECM. (**A**) Representative immunofluorescence images of Nucleostemin (NS, red; DAPI, grey) in TSPCs cultured under Blank, nECM, nECM−ΔBgn, and Bgn conditions. Scale bar, 200 μm. (**B**) Quantification of NS^+^ cell percentages (*n* = 15). (**C**) Relative mRNA expression of NS by RT-qPCR. (**D**) Relative mRNA expression of tenogenic markers Scx, Tnmd, Col1a1/Col3a1, and Lox1 (*n* = 3 or 4; outliers excluded).

Stemness. By day 7, the percentage of NS^+^ cells had significantly increased in both the Bgn and nECM groups, reaching levels comparable to each other and significantly higher than in the solvent control group (*p* < 0.0001). Compared with the solvent control group, the mRNA expression of NS was similarly elevated in both the nECM and Bgn groups, with no significant difference between them (Figure 6). These data suggest that Bgn supplementation maintains the stem cell phenotype of TSPCs at a level similar to that of the nECM group, indicating superior stem cell maintenance capacity compared with the solvent control group.

Tenogenic Differentiation. RT-qPCR analysis after 7 days showed that Bgn (1 μg/mL) significantly increased the expression of Col1a1, Scx, Tnmd and Lox1, while Col3a1 expression increased only slightly. This resulted in a substantial increase in the COL1A1/COL3A1 ratio. There was no statistically significant difference in these values compared to those in the nECM-supplemented group (*p* > 0.05). By contrast, the solvent control group exhibited minimal Col1a1 upregulation and a poor Col1a1/Col3a1 differentiation ratio (Figure 6).

**Figure 7:**
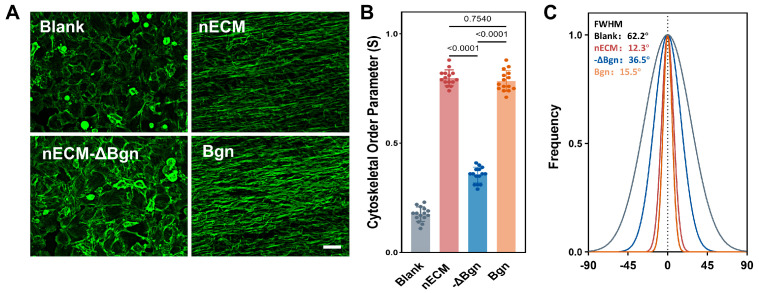
Bgn promotes anisotropic TSPCs alignment under cyclic tensile strain equivalently to nECM. (**A**) Representative F−actin (phalloidin, green) staining images of TSPCs cultured under Blank, nECM, nECM−ΔBgn, and Bgn conditions with cyclic uniaxial tensile strain. Scale bar, 200 μm. (**B**) Cytoskeletal Order Parameter (S) quantification (*n* = 15). (**C**) Directional frequency distribution histograms.

Anisotropic Alignment. The most significant functional difference observed among the groups was in the collective alignment of the cells. After seven days of culture under conditions of periodic uniaxial tensile strain (10%, 0.5 Hz, four hours daily), F-actin staining revealed that the cells in the Bgn and nECM groups exhibited dense, parallel, longitudinal alignment. This was in sharp contrast to the random orientation of the cells in the solvent control group. Quantitative analysis of orientation J confirmed this observation. Compared with the significantly reduced values in the solvent control group, the cohesion index values in both the Bgn and nECM groups were significantly higher and comparable (*p* < 0.0001). The orientation distribution histogram showed that the Bgn and nECM groups exhibited a near-uniaxial angular distribution confined to a narrow angular range. In contrast, the solvent control group displayed a uniform circular distribution, which is consistent with an absence of preferred orientation (Figure 7).

The biological performance of Bgn (1 μg/mL) was statistically equivalent to that of nECM (50 μg/mL) for all four endpoints, and significantly and consistently superior to the solvent control. This confirms that soluble recombinant Bgn is sufficient to reproduce the full nECM-mediated biological activity in TSPCs. To validate these functional findings, Bgn was efficiently immunodepletion from nECM with a removal rate of over 90%.

Compared to intact nECM, application of Bgn-depleted nECM (nECM-ΔBgn) at the same total protein concentration (50 μg/mL) significantly reduced all four functional endpoints: proliferation, stemness, tendon differentiation and anisotropic alignment were all decreased (all *p* < 0.001). Notably, for three of the four endpoints (proliferation, differentiation and alignment; all *p* < 0.05), the nECM-ΔBgn sample still exhibited a modest yet statistically significant advantage over solvent alone. This is consistent with the minor compensatory contributions of other nECM-enriched factors (including fibronectin, decapeptide-C and desmoglein), which remained in the Bgn-depleted fraction.

In summary, these converging gain-of-function and loss-of-function data establish that Bgn is the primary active molecule responsible for nECM bioactivity in TSPCs, and that its presence is both necessary and sufficient. These findings provide compelling evidence of the mechanism by which the biological effects observed with nECM supplementation are driven, showing that Bgn plays a primary role, while other ECM components play only a minor supporting one.

### 2.3. Bgn-Conditioned Cell Sheets Display Superior Pre-Organizational Architecture

**Figure 8:**
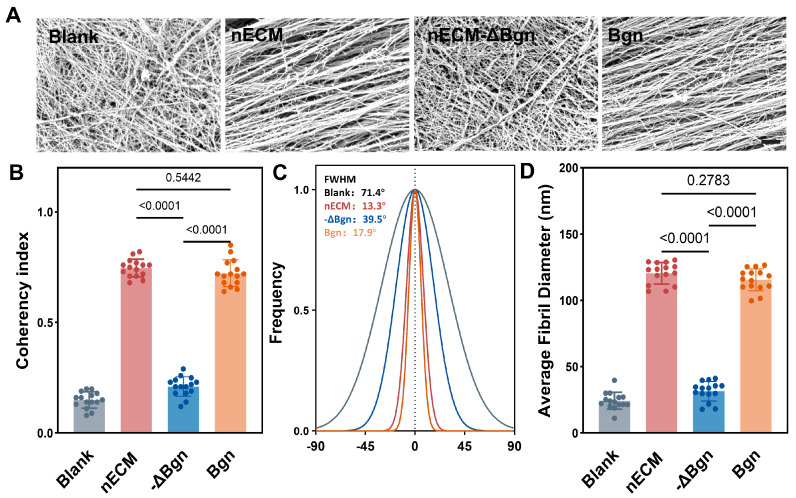
Bgn promotes anisotropic collagen fibril organization and maturation in TSPCs sheets. (**A**) Representative SEM images of ECM ultrastructure in cell sheets from Blank, nECM, nECM−ΔBgn, and Bgn groups. (**B**) Coherency index quantification of fibril alignment. (**C**) Fibril directional frequency distribution histograms. (**D**) Average fibril diameter (nm).

Under all supplementation conditions, the cell sheets formed over 14 days exhibited high and comparable survival rates upon harvest. This confirmed the good tolerability of the culture and harvest protocols across all conditions. SEM analysis of the ECM ultrastructure in the cell sections revealed significant differences in fiber organization among the groups (Figure 8). Bgn-treated sections (1 μg/mL) exhibited densely packed, highly parallel collagen fibers that were predominantly uniaxially oriented with a uniformity index of 0.72 ± 0.06. This closely resembled the structural organization of native rat Achilles tendons. nECM-treated cell sections (50 μg/mL) exhibited significant fiber alignment with a uniformity index of 0.75 ± 0.04 (compared to Bgn, *p* = 0.5442). This indicated a moderate degree of fiber organization. Bgn-depleted nECM sections (nECM-ΔBgn, 50 μg/mL) exhibited significantly reduced fiber alignment, with a consistency index of 0.21 ± 0.04 (compared to nECM, *p* < 0.0001). However, they were still significantly superior to the solvent control group, with a fiber diameter of 31.5 ± 7.4 nm and moderate uniformity (coefficient of variation: 23.42%). The solvent control group sections exhibited a markedly disordered fiber network approaching isotropic alignment (consistency index = 0.15 ± 0.04; *p* < 0.0001 compared to Bgn). Fiber diameter analysis showed that they were significantly larger than those in the solvent control group (24.3 ± 6.4 nm; *p* < 0.001) and exhibited significantly higher uniformity (coefficient of variation 7.15% vs. 26.22%), which is consistent with Bgn’s role in regulating lateral fiber fusion during collagen assembly.nECM sections exhibited larger fiber diameters (120.5 ± 8.2 nm) and higher uniformity (coefficient of variation 6.78%).

### 2.4. Bgn-Conditioned Cell Sheet Transplantation Achieves Near-Complete Structural and Functional Tendon Regeneration In Vivo

**Figure 9:**
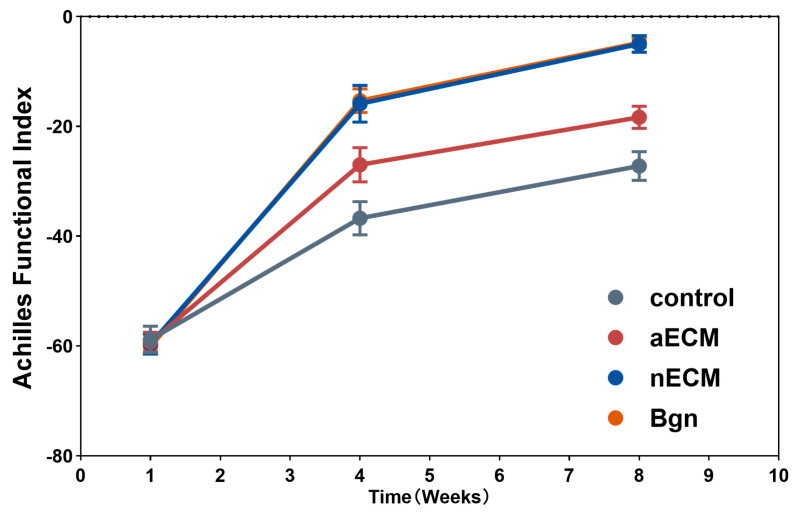
Bgn and nECM implantation accelerate functional gait recovery after Achilles tendon injury. Achilles Functional Index (AFI) measured at postoperative weeks 1, 4, and 8 in control, aECM, nECM, and Bgn groups, *n* = 8 per group.

Functional gait recovery (AFI). One week after surgery, all four groups exhibited severe functional deficits and comparable AFI values, confirming equivalent injury severity across groups. During the subsequent recovery period, from week 1 to week 8, the treatment groups exhibited markedly different recovery trajectories. The nECM group showed the most significant recovery, demonstrating marked functional improvement by week 4, which continued to progress until week 8. By this time, they had achieved the highest degree of recovery, approaching normal function. The aECM and Bgn groups exhibited moderate recovery with similar improvement patterns, but both were significantly inferior to the nECM group. The control group showed the slowest recovery, with significant functional deficits persisting throughout the observation period. AFI values remained relatively high at week 8, indicating ongoing functional impairment (intergroup difference, *p* < 0.0001). Footprint morphometric analysis confirmed that only the nECM group achieved bilateral symmetry in stride length, toe spread and toe spacing, while the other groups retained significant asymmetry (Figure 9).

**Figure 10:**
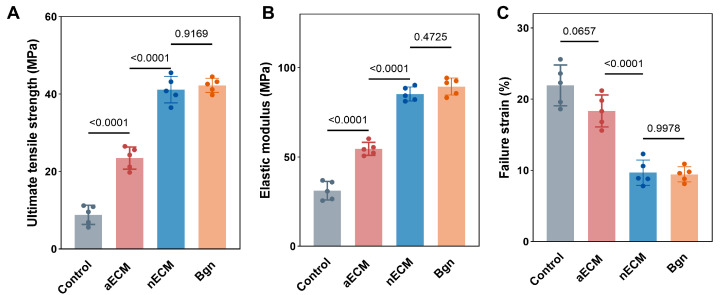
Biomechanical properties of regenerated tendons at 8 weeks post-surgery. (**A**) Ultimate tensile strength (UTS), (**B**) elastic modulus, and (**C**) failure strain of regenerated tendons in the Control, aECM, nECM, and Bgn groups (*n* = 5).

Biomechanical properties. The biomechanical properties of the regenerated tendons were evaluated using tensile testing eight weeks after surgery. In terms of ultimate tensile strength (UTS), both the Bgn and nECM groups exhibited significantly higher values than the aECM and control groups (*p* < 0.0001). Interestingly, no significant difference was observed between the Bgn and nECM groups (*p* = 0.9169), suggesting that they had comparable levels of tensile strength recovery. The UTS values for the Bgn and nECM groups were close to those of an undamaged native tendon, suggesting that their load-bearing capacity had almost fully recovered. The recovery trend for elastic modulus was similar. The elastic modulus values for the Bgn and nECM groups were significantly higher than those of the aECM group. There was also no statistically significant difference between the Bgn and nECM groups (*p* = 0.4725). This suggests that both bio-reinforced ECM scaffolds substantially recovered tissue stiffness and structural integrity, reflecting improved resistance to deformation under mechanical loading. Regarding failure strain, the Bgn and nECM groups exhibited significantly lower values than the control group (*p* < 0.0001), reflecting the increased structural stiffness characteristic of mature collagen tissue. There was no significant difference between the Bgn and nECM groups (*p* = 0.9978), which further confirms their equivalent efficacy in promoting ordered tissue remodeling and reducing tissue elasticity to levels approaching those of native tendons (Figure 10). Overall, biomechanical analysis indicates that both Bgn and nECM treatments achieved excellent, comparable restoration of mechanical properties, significantly outperforming the aECM group and closely mimicking the functional characteristics of native tendon tissue (Figure 10).

**Figure 11:**
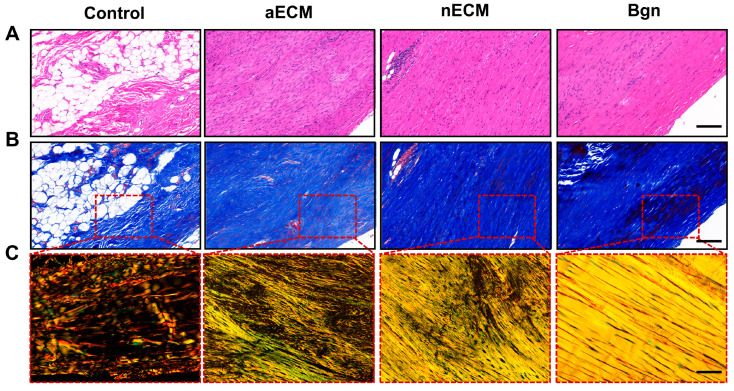
Histological analysis of regenerated tendons at 8 weeks post−surgery. (**A**) Hematoxylin and eosin (H&E) staining showing overall tissue morphology and cell arrangement. Scale bar, 200 μm. (**B**) Masson’s trichrome staining illustrating collagen deposition and maturation. Red dashed boxes indicate regions shown at higher magnification in (**C**). Scale bar, 200 μm. (**C**) Polarized light microscopy of Sirius red−stained sections revealing collagen fiber alignment and anisotropy. Scale bar, 100 μm.

Histological structure. By week 8, significant treatment-dependent differences in the microstructural characteristics of the repaired tendon tissue had emerged. Tendons repaired with Bgn exhibited an ordered collagen structure and tendon cell morphology indistinguishable from normal tendons, indicating that the tissue structure had approached a physiologically normal state. Similarly, tendons repaired with nECM had a comparable collagen tissue structure and level of maturity to those repaired with Bgn, and both treatments were significantly superior to the aECM and solvent control groups at the microscopic level. By contrast, the solvent control group exhibited a markedly disorganized, scar-like extracellular matrix with disordered collagen fiber arrangement, abnormal cell morphology and typical scar characteristics (Figure 11A). The aECM group exhibited an intermediate phenotype: although its collagen tissue structure and maturity were inferior to those of the Bgn and nECM groups, they were superior to those of the solvent control group.

Masson’s trichrome staining was used to quantify collagen deposition and maturation status. In the Bgn and nECM groups, red-stained areas representing mature collagen fibers were widely distributed and dense, indicating sufficient collagen deposition and fiber maturation comparable to normal tendons. In contrast, the aECM group exhibited relatively sparse red-stained areas, reflecting incomplete collagen deposition. The solvent control group showed a marked absence of red-stained areas and had fine, highly disorganized fibers. This indicates severely impaired collagen deposition and an increased proportion of scar tissue (Figure 11B). These distinct characteristics confirm the superiority of Bgn and nECM in promoting tissue reconstruction and collagen maturation.

Polarized light microscopy of Sirius red-stained fibers revealed that the Bgn and nECM groups exhibited similar coherence indices and anisotropy patterns. Both groups demonstrated high fiber orientation and coherence, indicating that the microstructure of the repaired tissue was approaching an ordered, regular physiological state. In contrast, the aECM group exhibited intermediate levels of coherence and anisotropy. While its fiber organization was superior to that of the control group, it had not yet reached the maturity level of the Bgn/nECM groups. Meanwhile, the solvent control group showed a significantly reduced coherence index and anisotropy, with fibers remaining in a highly disordered state and lacking distinct orientation (Figure 11C). In summary, comprehensive microscopic observations reveal that tendons repaired with Bgn and nECM demonstrate significant advantages in terms of tissue structural remodeling, collagen maturation and deposition, and ordered fiber arrangement. These results suggest that by week 8, both treatment strategies demonstrate comparable biological efficacy in promoting tendon regeneration.

## 3. Discussion

### 3.1. nECM Defines an Age-Dependent Regenerative Niche

Adult tendons heal primarily through repair, whereas neonatal tendons have an extraordinary capacity for nearly scar-free regeneration [23]. The results of this study suggest that this age-related difference in healing quality is mechanistically related to the composition of the ECM rather than being an irreversible consequence of developmental stage [24]. Specifically, nECM significantly enhances the proliferation and maintenance of stemness of TSPCs, as well as their differentiation and anisotropic alignment, whereas adult ECM fails to improve any of these parameters relative to the solvent control group across all experimental assays and time points. Secondly, delivering soluble factors from nECM exogenously is sufficient to restore the regenerative behavior of TSPCs in adult cell populations. Our data provide functional evidence that this correlation is causal, at least in part, and support the broader therapeutic theory that recreating the composition of nECM will steer adult tendon healing towards regeneration rather than scar formation.

### 3.2. Biglycan Is the Principal Bioactive Determinant of nECM

Previous studies have shown that SLRPs undergo dynamic remodeling during tendon ECM maturation, and their levels in adult tissues decline sharply. This developmental expression pattern establishes Bgn as a potential mediator of nECM bioactivity. Immunofluorescence analysis in this study confirmed the uniform distribution of strong Bgn immunoreactivity in the interstitial spaces of neonatal tendons. The progressive loss of other key microenvironmental components and the age-dependent decline in Bgn protein abundance provide correlative evidence linking Bgn availability to regenerative potential.

Genetic evidence from Bgn knockout mice is important for interpreting the results of this study [25,26]. The tendons and ligaments of these animals exhibit disorganized collagen fiber alignment and uneven diameter distribution, reduced bone mineral density, and a progressive connective tissue phenotype with features resembling several manifestations of Ehlers–Danlos syndrome. This genetic loss-of-function has been further supported by this study. Supplementation with recombinant Bgn reproduced all four functional enhancement effects of TSPCs driven by native extracellular matrix (nECM)—proliferation, stem cell properties, tendon differentiation, and anisotropic alignment. Conversely, immunodepletion of Bgn from nECM reduced all four functional parameters. This converging, bidirectional evidence confirms that Bgn is a major determinant that accounts for much of the biological activity of nECM.

The broad effects of Bgn on the four distinct functional endpoints of TSPCs suggest that its mechanism of action involves multiple, complementary signaling pathways rather than a single, linear one. As a collagen fiber-associated SLRP that bears chondroitin sulfate/dermatan sulfate glycosaminoglycan chains, Bgn is structurally positioned at the interface between the fibrillar collagen network and the pericellular microenvironment. This enables the regulation of integrin-mediated outside-in signaling. It is known that the major collagen-binding integrin α11β1 in tendon fibroblasts mediates cellular alignment with, and force transduction from, the fibrillar collagen matrix. Bgn’s tight binding to collagen fibers makes it a plausible regulator of integrin binding and aggregation, providing a mechanistic basis for the anisotropic collective alignment observed in Bgn-treated cultures [6]. In Bgn-treated culture systems, maintenance of the stem cell marker is consistent with enhanced tendon differentiation capacity, rather than being inconsistent with it. This is consistent with the concept of a pre-activated progenitor cell state. This dual function of maintaining the progenitor pool while guiding differentiation is a hallmark of the regenerative microenvironment. This is in stark contrast to the adult injury environment, where TSPCs are rapidly depleted due to premature differentiation or are directed towards fibrotic, osteogenic or chondrogenic fates.

The temporal dynamics of Bgn activity are also important to consider. Our results suggest that Bgn may exert stage-dependent functions during tendon regeneration. During the early phase of repair, Bgn appears to support TSPC proliferation and preserve progenitor-associated features, thereby maintaining an adequate pool of repair-responsive cells. At the same time, the upregulation of tenogenic markers such as Scx and Tnmd suggests that Bgn also facilitates early tenogenic commitment. During the subsequent matrix maturation phase, Bgn may act more prominently as a structural regulator by interacting with collagen fibrils and modulating fibril fusion, diameter control, and anisotropic matrix organization. Therefore, Bgn may not act exclusively at a single stage but may instead coordinate early cellular commitment with later extracellular matrix maturation.

### 3.3. Bgn-Conditioned Cell Sheets Achieve Near-Normal Tendon Regeneration In Vivo

The transplantation of Bgn-conditioned TSPCs cell sheets into full-thickness defects in the Achilles tendons of rats produced compelling outcomes at eight weeks post-surgery that were consistent across functional, biomechanical, and histological assessments. The Achilles Functional Index (AFI) scores in the Bgn group were statistically indistinguishable from those of the uninjured contralateral limb, indicating near-complete restoration of gait mechanics—a clinically meaningful outcome integrating the contributions of tendon mechanical integrity, proprioceptive feedback and neuromuscular coordination.

Biomechanically, Bgn-repaired tendons achieved ~80% recovery of ultimate tensile load and approached normal stiffness values, significantly outperforming both the aECM and vehicle control groups. The strain at failure in the Bgn group was significantly lower than in the control group. Importantly, no significant differences were detected between the Bgn and nECM groups across any biomechanical parameter, indicating that a single recombinant molecule can recapitulate the mechanical benefits of the entire nECM extract. This finding has significant translational implications as a defined, single-molecule therapeutic is inherently more reproducible, scalable and amenable to regulatory approval than a complex tissue-derived extract.

Histologically, Bgn-repaired tendons displayed well-organized, parallel collagen architecture, tenocyte morphology indistinguishable from that of native tendons, strong type I collagen predominance with minimal type III collagen, and a mature collagen composition indicative of ordered tendon rather than fibrotic scarring. Analysis of collagen fibril diameter revealed appropriate mean fibril size and substantially greater diameter uniformity in the Bgn group compared to controls, consistent with Bgn’s established role in regulating lateral fibril fusion and diameter control during collagen fibrillogenesis [27].

These superior in vivo outcomes are directly attributable to the pre-organized architecture of Bgn-conditioned cell sheets prior to transplantation. Unlike dissociated cell suspensions or randomly organized scaffolds, Bgn-conditioned cell sheets exhibited anisotropic cell alignment, organized collagen fibril deposition and established cell–cell and cell–matrix interactions at the time of implantation. This pre-existing architectural template likely served as a structural and instructive foundation for subsequent in vivo remodeling. This finding supports the broader principle in tissue engineering that ECM-level preorganization of an engineered graft translates into superior regenerative outcomes in vivo a rationale aligned with efforts across multiple musculoskeletal tissues to recapitulate native tissue architecture through biomaterial design, electrospun scaffolds, and bioprinting strategies [28]. The cell sheet approach employed here offers particular advantages as it preserves the endogenous pericellular matrix and intercellular junctions formed during culture, providing a transplantable, scaffold-free construct that avoids the potential complications of exogenous biomaterial degradation products and foreign body responses.

### 3.4. Limitations and Future Directions

This study has several limitations that warrant attention. Firstly, the study was conducted exclusively in rat models, so its applicability to large animals or human-scale defects has yet to be validated. While the eight-week observation period reflects early structural recovery, it does not evaluate long-term mechanical durability or the risk of late-stage re-rupture. The study did not directly investigate the downstream signaling pathways mediating Bgn’s action. Future research should employ integrin-blocking antibodies or pathway-specific inhibitors to elucidate these mechanisms. Conducting longitudinal large-animal studies is an essential prerequisite for advancing to clinical translation.

From a translational perspective, the Bgn-functionalized cell sheet strategy has several potential advantages. Because recombinant Bgn is a defined single-molecule extracellular matrix component, it may offer greater reproducibility and regulatory clarity than complex tissue-derived matrix extracts. The cell sheet preparation process is also compatible with scalable culture systems and could potentially be adapted to Good Manufacturing Practice (GMP) conditions. Regarding cell sources, autologous TSPCs may reduce immunogenicity but could be limited by donor age, tissue availability, and patient-to-patient variability. In contrast, allogeneic TSPCs from young healthy donors may provide a more standardized and scalable source, although their immunological safety and long-term integration require further evaluation. Future large-animal studies will be needed to assess manufacturing feasibility, safety, durability, and regulatory requirements before clinical translation.

## 4. Materials and Methods

### 4.1. Animals and Ethics

All animal experiments were approved by the Animal Experiment Ethics Committee of the Peking University Health Science Centre (approval no. DLASBD0766, 27 December 2024). Male Sprague-Dawley (SD) rats were purchased from Beijing Vital River Laboratory Animal Technology Co., Ltd., Beijing, China. Only male rats were used to minimize potential confounding effects caused by estrous cycle-associated hormonal fluctuations, which may influence collagen metabolism, matrix remodeling, inflammatory responses, and tendon biomechanical properties. Two age groups were used in the experiments: newborn pups (postnatal days 3–5; abbreviated as P3–P5) and skeletally mature adult rats (12 weeks old and weighing 250–280 g). All animals were housed under specific pathogen-free (SPF) conditions. They had free access to standard rodent chow and water. Prior to tissue collection, the animals were euthanized with 3% isoflurane and maintained at a concentration of 1.5–2%.

### 4.2. Preparation of Decellularized Tendon ECM

Immediately after euthanasia, the bilateral Achilles tendons were aseptically harvested in a sterile laminar flow hood. The surrounding tendon sheath, adipose tissue and fibrocartilage at the calcaneal insertion were carefully removed. The tendon tissue was stored at −80 °C until processing. To prepare decellularized scaffolds, we subjected tendon samples to three cycles of freezing and thawing, followed by agitation in a 0.1% SDS solution for 24 h with continuous orbital shaking at 4 °C; followed by thorough washing with PBS for a total of 12 h with the medium replaced every 3 h to remove residual detergent [29,30]. The decellularized scaffolds were freeze-dried for 48 h and stored at −80 °C in a dry environment.

### 4.3. ECM Protein Extraction

Lyophilized ECM scaffolds were first pulverized into a fine powder under liquid nitrogen using a cryogenic grinder (abclonal, Wuhan, China). For protein extraction, the powdered ECM was resuspended in a denaturing buffer (8 M urea, 0.5 M guanidine chloride, 50 mM Tris-HCl [pH 7.4], 10 mM EDTA, and 1× protease inhibitor cocktail) at a 1:20 (*w*/*v*) ratio under continuous stirring at 4 °C for 12 h. After centrifugation (13,000× *g*, 30 min, 4 °C), the supernatant was collected and subjected to stepwise dialysis using a 3.5 kDa membrane to remove denaturants and promote refolding. The denaturant concentration was gradually decreased in the presence of a redox pair (GSSG/GSH). Specifically, the extract underwent sequential dialysis: (i) 12 h in 50 mM Tris-HCl (pH 8.0) containing 6 M urea, 0.5 mM GSSG, and 5 mM GSH; (ii) 12 h in the same buffer with 2 M urea; and (iii) 48 h in PBS (pH 7.4) at 4 °C with buffer changes every 12 h. The final dialysate was concentrated via BCA assay, then lyophilized and stored at −80 °C.

### 4.4. Immunofluorescence Confirmation of Bgn Expression In Situ

The sections were fixed with 4% paraformaldehyde (PFA) for 10 min, permeabilized with a solution of 0.3% Triton X-100 in PBS for 15 min and blocked at room temperature with a solution of 5% normal goat serum in PBS for one hour. Primary antibody incubation: Rabbit anti-Bgn (1:200; 16409-1-AP, Proteintech, Wuhan, China) was incubated 12 h at 4 °C. The secondary antibody used was Alexa Fluor 568-labeled goat anti-rabbit IgG (1:500; ab175471, Abcam, Cambridge, UK) for 1 h at room temperature. Cell nuclei were counterstained with DAPI (1 μg/mL).

### 4.5. Isolation and Culture of TSPCs

We harvested Achilles tendons from 4-week-old male SD rats and processed them for TSPCs isolation via enzymatic digestion. Briefly, tendons were minced into approximately 1 mm^3^ pieces and incubated in DMEM supplemented with 1 mg/mL type I collagenase (Worthington) at 37 °C under constant agitation for 8 h. The digestion supernatant was then filtered through a 70 µm cell strainer, centrifuged at 500× *g* for 5 min and washed twice with PBS. To enrich the TSPCs, the cells were seeded at a low density of 100 cells/cm^2^ and cultured for 7–10 days under standard conditions (37 °C) in complete medium. Only wells that formed distinct clusters were passaged. All experiments used TSPCs at passage numbers 2–4 [13].

### 4.6. Biglycan Immunodepletion

Bgn immunodepletion procedure for removing Bgn from nECM is as follows: Use 5 μg of rabbit anti-Bgn antibody (16409-1-AP, Proteintech, Wuhan, China) per milligram of total nECM protein, and conjugate it with Protein G–Dynabeads (Bimake). The antibody-conjugated magnetic beads were incubated with the nECM solution under continuous rotation at 4 °C for 12 h; the beads were recovered using a magnetic separator (Bimake, Houston, TX, USA), and the supernatant (nECM-ΔBgn) was retained.

### 4.7. Cell Proliferation Assays

For the EdU uptake assay, TSPCs were cultured in supplemented medium for 44 h, followed by a 4 h pulse treatment with EdU (10 μM). The cells were then fixed (4% PFA, 15 min), permeabilized (0.5% Triton X-100, 10 min), and subjected to a Click-iT reaction (Alexa Fluor 568-azide, 30 min, room temperature), followed by counterstaining with DAPI. For proliferation kinetics, cells were seeded into 96-well plates (Corning, NY, USA) (5 × 10^3^ cells per well), and CCK-8 reagent (1:10 dilution) was added at days 1, 3, 5, and 7; after incubation at 37 °C for 2 h, absorbance was measured at 450 nm (*n* = 6 wells per group at each time point).

### 4.8. Stemness Assessment

After 7 days of culture, stemness was assessed by immunofluorescence detection of nucleostemin (NS). Cells were fixed (4% PFA, 20 min), permeabilized (0.3% PBST, 20 min), and blocked (5% BSA/PBS, 1 h). Incubation with a rabbit anti-nucleostemin primary antibody (1:200; Abcam ab70346) at 4 °C overnight, followed by incubation with Alexa Fluor 568-labeled goat anti-rabbit IgG secondary antibody (1:500, 1 h, room temperature). Cell nuclei were counterstained with DAPI. Nucleostemin mRNA expression was additionally evaluated by RT-qPCR, and the corresponding primer sequences are provided in Table 1.

### 4.9. Tenogenic Differentiation

After 7 days of supplementation, tenogenic differentiation was assessed at the mRNA and protein levels. For RT-qPCR, total RNA was extracted, and reverse transcription was performed. Real-time quantitative PCR was performed with the following cycling conditions: 95 °C for 30 s; 95 °C for 5 s and 60 °C for 30 s, for a total of 40 cycles, followed by melting curve analysis. Target genes included Col1a1, Col3a1, Scx (Scleraxis), Tnmd (Tenomodulin), and Lox1 (Lysyl oxidase 1) [31,32]. Primer sequences are provided in Table 1. The COL1A1/COL3A1 ratio was used as the primary indicator of collagen maturity [11].

### 4.10. Cell Morphology and Anisotropic Alignment Analysis

Cells were cultured on BioFlex^®^ six-well plates (Flexcell International Corporation, Burlington, NC, USA) coated with type I collagen. Cyclic uniaxial tensile strain (10%, 0.5 Hz, for 4 h daily over 7 consecutive days) was applied. The culture medium was changed daily after each loading session. After 7 days, cells were fixed with 4% PFA (15 min), permeabilized with 0.2% Triton X-100 (10 min), and stained with FITC-phalloidin (1:200). Confocal images were acquired at 20× magnification. The orientation of the F-actin cytoskeleton was analyzed using the OrientationJ plugin 2.0.7 (Fiji/ImageJ 1.54p).

### 4.11. Cell Sheet Fabrication and Characterization

TSPCs were seeded at high density (2 × 10^4^ cells/cm^2^) onto BioFlex culture plates in culture medium supplemented with the corresponding treatment conditions (Bgn 1 μg/mL, nECM 50 μg/mL, aECM 50 μg/mL, or solvent), along with 50 μg/mL ascorbic acid (Sigma-Aldrich) to stimulate endogenous ECM synthesis [33]. Throughout the 14-day culture period, mechanical stimulation was applied: 10% uniaxial stretching at a frequency of 0.5 Hz for 4 h daily. The culture medium was changed every 48 h to maintain the culture. Cell sheets were harvested according to a dissection protocol by performing gentle mechanical dissection at the membrane-medium interface using a cell scraper. Samples were fixed in 2.5% glutaraldehyde buffer at 4 °C for 4 h, dehydrated using an ethanol gradient, subjected to critical-point drying, and coated with platinum by sputtering. SEM images were acquired using a JSM-7900F field-emission scanning electron microscope (JEOL Ltd., Tokyo, Japan).

### 4.12. In Vivo Rat Achilles Tendon Full-Thickness Defect Model

Male SD rats (12 weeks old, weighing 250–280 g) were obtained from Beijing Vital River Laboratory Animal Technology Co., Ltd. Animals were housed under SPF conditions with free access to standard rodent chow and water. The sample size of *n* = 8 per group was determined based on a power analysis (α = 0.05, power = 0.8) to detect a 30% difference in biomechanical properties, consistent with previous tendon defect studies [34]. For the in vivo Achilles tendon full-thickness defect experiment, *n* = 8 rats per group were used. Animals were randomly allocated to treatment groups (Control, aECM, nECM, Bgn) to minimize bias related to cage location or order of surgery.

Prior to surgery, anesthesia was induced with 3% isoflurane and maintained at a concentration of 1.5–2% via a nasal mask. Under sterile conditions, the right Achilles tendon was exposed via a 2 cm posterior longitudinal incision. A full-thickness transection was performed at the midpoint of the tendon using microsurgical scissors. After confirming the proximal and distal tendon stumps, the stumps were approximated using a modified Kessler suture technique with 4-0 PDS, followed by continuous superficial tendon suturing with 6-0 Prolene [34]. Post-operative analgesia (Carprofen, 5 mg/kg, subcutaneously) was administered immediately after surgery and daily for the first 48 h to minimize pain and distress. Animals were monitored daily for signs of infection, wound dehiscence, or impaired mobility.

At the designated endpoint (8 weeks post-surgery), animals were deeply anesthetized with 3% isoflurane and euthanized by cervical dislocation prior to tissue harvest. Subsequently, the entire right Achilles tendon-calcaneus-gastrocnemius complex was excised for subsequent biomechanical testing and histological analysis. All outcome assessments, including gait analysis (AFI), biomechanical testing, and histological scoring, were performed by investigators blinded to group allocation.

### 4.13. Achilles Functional Index Assessment

Functional recovery was monitored longitudinally at 1, 4, and 8 weeks post-surgery using the Achilles Tendon Function Index (AFI), a validated rodent gait analysis metric based on standardized footprint analysis. After applying ink to the plantar surfaces of both hind feet, the animals were allowed to walk freely on a paper-lined walkway (40 × 8 cm). At each time point, three consecutive and uninterrupted gait cycles were recorded for each animal, and the two most complete cycles were selected for analysis [35].

### 4.14. Biomechanical Testing

Euthanasia was performed after 8 weeks, and the Achilles tendon-calcaneus complex was carefully dissected from the surrounding soft tissues. The calcaneus was embedded in dental resin, and the proximal tendon of the gastrocnemius muscle was clamped using specially designed serrated forceps. The gauge length (between the clamping surfaces) was standardized to 10 mm. Throughout the preparation process, all specimens were kept moist with gauze soaked in PBS. Uniaxial tensile testing was performed using a servo-controlled materials testing machine (Instron, Norwood, MA, USA). The loading protocol included: (i) preconditioning (10 cycles, 0–1 N, 0.1 Hz), (ii) 5 min rest, (iii) progressive loading to failure (crosshead displacement rate of 1 mm/min). The ultimate tensile load (N), stiffness (N/mm), and fracture displacement (mm) were extracted from each curve. Mechanical recovery was expressed as a percentage of the value from the contralateral normal limb of each animal.

### 4.15. Histological and Immunohistochemical Analysis

Mid-section tendon tissue was fixed in 4% paraformaldehyde (PFA) for 24 h, paraffin-embedded, and sectioned longitudinally into 5-μm-thick sections. Paraffin sections were stained as follows: (i) H&E staining; (ii) Masson’s trichrome staining; and (iii) Sirius red staining (0.1% saturated picric acid solution).

### 4.16. Statistical Analysis

Quantitative data are expressed as the mean ± standard deviation (SD). Prior to analysis, data normality and homogeneity of variances were verified using the Shapiro–Wilk and Levene’s tests, respectively. Intergroup comparisons were performed using Student’s *t*-test for two-group analyses or one-way ANOVA with Tukey’s HSD post hoc test for multi-group comparisons. All statistical procedures were executed using GraphPad Prism version 10.0, with statistical significance defined as a two-tailed *p*-value < 0.05; precise *p*-values are indicated where applicable.

## 5. Conclusions

In summary, this study suggests that the postnatal decline of Bgn in the adult tendon matrix contributes to the fibrotic repair phenotype. By adding Bgn signaling to a scaffold-free TSPCs sheet platform, we successfully created a 3D engineered microenvironment that mimics the neonatal extracellular matrix. This approach overcomes the limitations of traditional 2D culture systems by enabling the investigation of complex paracrine signaling and extracellular matrix remodeling processes within a physiologically relevant environment. Bgn-regulated cell sheets provide a structural template for regeneration and deliver critical molecular signals for tendon differentiation and organization of an anisotropic matrix. This work sheds light on the mechanisms involved in the determination of stem cell fate by the ECM and establishes Bgn-functionalized cell sheet therapy as a translatable, scaffold-free strategy for overcoming fibrotic repair and restoring functional tendon architecture. Future genomic and proteomic profiling of Bgn-conditioned TSPCs should focus on defining specific downstream pathways, including TGF-β/Smad modulation, integrin-mediated mechanotransduction, FAK–ERK signaling, and the broader SLRP interaction network involving decorin, fibromodulin, and lumican. Such multi-omics analyses will help clarify the ECM-driven signaling networks and gene regulatory cascades that underlie tenogenic commitment, matrix organization, and functional tendon restoration.

## Figures and Tables

**Table 1:** Primer sequences used for RT-qPCR.

GENE	Forward (5′-3′)	Reverse (5′-3′)
Scx	AGAACACCCAGCCCAAACAG	TCACGGTCTTTGCTCAACTTTC
Tnmd	CAGAGAACTGTGAGGGCTGTC	CTCCATGCCATAGGTCTTCTTGG
Lox1	TACTTCCAGTACGGTCTCCCG	CGTAGCAGTACCCTGTGGTC
Col1a1	CACTGCAAGAACAGCGTAGC	AGTTCCGGTGTGACTCGTG
Col3a1	TGGGCCTCAAGGTGTAAAGG	GCCCTGGATTACCATTGTTGC
NS	TGCACACAGCATACAAGTCCT	TGACTCTTCGGGGATGTCCT

## Data Availability

The original contributions presented in the study are included in the article. Further inquiries can be directed to the corresponding authors.

## Abbreviations

The following abbreviations are used in this manuscript:

aECM	Adult extracellular matrix
AFI	Achilles Functional Index
Bgn	Biglycan
BMP	Bone morphogenetic protein
CCK-8	Cell Counting Kit-8
ECM	Extracellular matrix
EdU	5-ethynyl-2′-deoxyuridine
FAK	Focal adhesion kinase
GAG	Glycosaminoglycan
H&E	Hematoxylin and eosin
nECM	Neonatal extracellular matrix
RT-qPCR	Reverse transcription quantitative polymerase chain reaction
Scx	Scleraxis
SEM	Scanning electron microscopy
SLRP	Small leucine-rich proteoglycan
TGF-β	Transforming growth factor-beta
Tnmd	Tenomodulin
TSPCs	Tendon stem/progenitor cells
